# Health-Related Factors Associated with Mode of Travel to Work

**DOI:** 10.1155/2013/242383

**Published:** 2013-02-27

**Authors:** Melissa Bopp, Andrew T. Kaczynski, Matthew E. Campbell

**Affiliations:** ^1^Department of Kinesiology, The Pennsylvania State University, 268R Recreation Building, University Park, PA 16802, USA; ^2^Department of Health Promotion, Education and Behavior, Arnold School of Public Health, Prevention Research Center, University of South Carolina, Columbia, SC 29208, USA

## Abstract

Active commuting (AC) to the workplace is a potential strategy for incorporating physical activity into daily life and is associated with health benefits. This study examined the association between health-related factors and mode of travel to the workplace. *Methods*. A volunteer convenience sample of employed adults completed an online survey regarding demographics, health-related factors, and the number of times/week walking, biking, driving, and using public transit to work (dichotomized as no walk/bike/drive/PT and walk/bike/drive/PT 1 + *x*/week). Logistic regression was used to predict the likelihood of each mode of transport and meeting PA recommendations from AC according to demographics and health-related factors. *Results*. The sample (*n* = 1175) was aged 43.5 ± 11.4 years and was primarily White (92.7%) and female (67.9%). Respondents reported walking (7.3%), biking (14.4%), taking public transit (20.3%), and driving (78.3%) to work at least one time/week. Among those reporting AC, 9.6% met PA recommendations from AC alone. Mode of travel to work was associated with several demographic and health-related factors, including age, number of chronic diseases, weight status, and AC beliefs. *Discussion*. Mode of transportation to the workplace and health-related factors such as disease or weight status should be considered in future interventions targeting AC.

## 1. Introduction

The economic cost of preventable chronic disease in the United States is substantial, with the direct and indirect costs associated with cancer, cardiovascular disease, diabetes, mental health disorders, and pulmonary conditions estimated at more than $1 trillion for the general population in 2003. Among employed adults, much of this economic burden is shouldered by employers in terms of private health insurance expenditures and lost productivity, with the costs associated with chronic disease nearing $465 billion [1]. The visionary initiative targeting population level health is found in the US Department of Health and Human Services' Healthy People 2020 and includes goals of attaining high-quality, longer lives free of preventable disease and premature death [2]. This document includes goals and objectives focused on changing health behaviors that contribute to chronic disease morbidity and mortality, including specifically improving rates of physical activity participation along with environmental and policy approaches aimed at supporting this behavior across the lifespan.

Evidence outlining the benefits of regular physical activity participation for the prevention of chronic disease and premature mortality is substantial [3, 4]. Epidemiological and clinical trials have documented the benefits of physical activity in preventing diabetes and metabolic disorders [5–8], cardiovascular disease [9–11], certain cancers [12–16], and mental health disorders [17–19]. The majority of these studies include data on all forms of physical activity (leisuretime, occupational, and transportation related). When specifically examining the health effects of transportation-related physical activity to work, known as active commuting (AC), data from epidemiological surveys have found relationships between active travel and a lesser presence of self-reported obesity [20–22] and a reduced risk of cardiovascular disease and all-cause mortality [23–26]. Despite these known benefits of active transport, in the United States, rates of AC remain low (3% reporting walking to work, <1% report biking to work), especially in comparison to other countries (e.g., The Netherlands: 25% of trips are made by bicycle) [27–29].

Recent research has addressed individual, social, and environmental factors associated with AC to work. Some documented correlates of AC include demographics (age, gender, income, and race/ethnicity) [30, 31], psychosocial (self-efficacy, behavioral beliefs, attitudes, and intention) [32–35], and environmental influences (traffic, walkable and bikeable features, safety, and convenient public transport close to the workplace) [36–41]. However, few, if any, studies have focused in much depth on how diverse health-related factors are associated with AC [42], despite the acknowledgment that health is a prime determinant, motivator, and outcome of AC [25]. Moreover, the impact of health-related influences on specific modes of travel (e.g., walking, biking, transit, and driving) has received even less attention. Additionally, few studies have explored the extent to which AC provides sufficient opportunity to achieve recommended levels of physical activity that are adequate to achieve health benefits [43–45]. Moreover, to the extent that this is possible, what are the characteristics of individuals who engage in enough AC to meet physical activity recommendations?

Given these considerations, the purpose of this study was twofold. Our primary aim was to examine the relationship between numerous demographic and health-related factors and mode of travel to work (walking, biking, driving, and public transit). The secondary aim of the study was to examine the health-related factors associated with achieving current public health recommended levels of physical activity [46] via AC.

## 2. Methods

### 2.1. Survey Design

This cross-sectional survey was delivered online from June to December 2011 using Qualtrics (Provo, UT) and was approved by the Pennsylvania State University Institutional Review Board.

### 2.2. Participants and Recruitment

**Figure 1:**
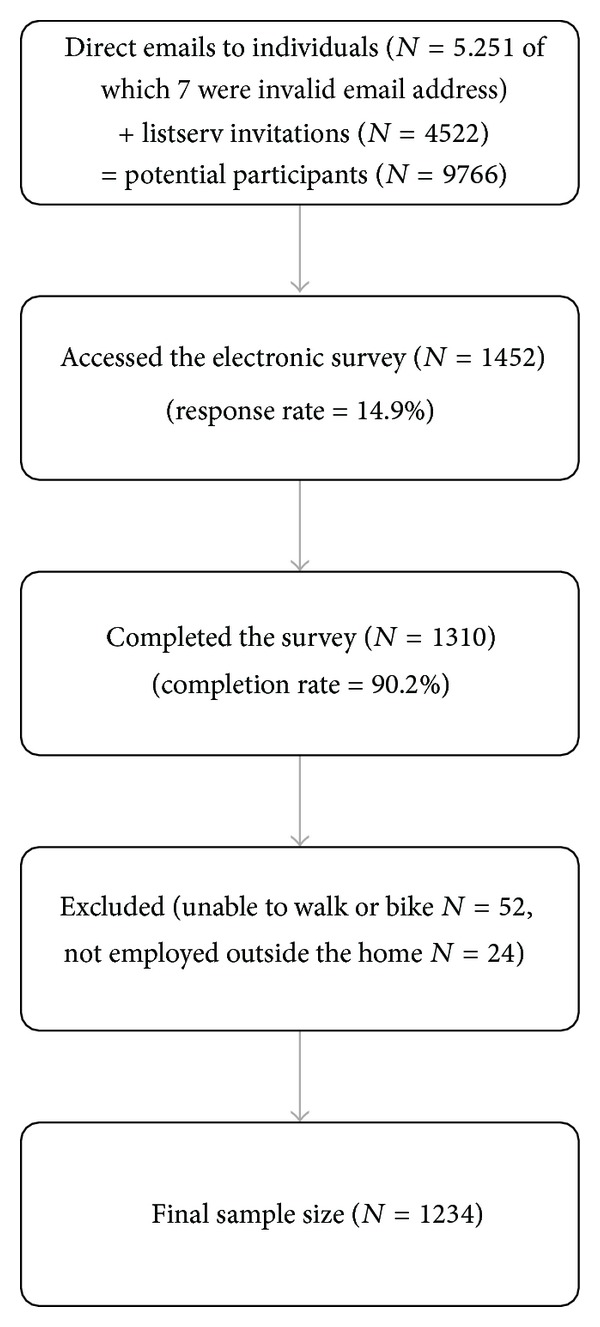
Participant recruitment.

To be eligible, participants had to be over the age of 18 years, employed full- or part-time outside of the home, and physically able to walk or bike. Recruitment was focused in the mid-Atlantic region of the USA (PA, OH, WV, MD, NJ, and DE). The primary recruitment strategy involved visiting the websites of large employers (e.g., K-12 school districts, local/county government, private businesses, and universities/colleges) in medium to large cities for employee email addresses and subsequently contacting the employees directly with an email invitation. In cases where employee email addresses were not available, we contacted employers directly and asked them to distribute an electronic invitation to participate in the survey via listserv, e-newsletter, or mass email to their employees. Among employers contacted directly (*n* = 142), two employers refused to send out an email invitation, 84 did not respond in any way, and 56 sent out a recruitment invitation. Recruitment of participants is displayed in Figure 1.

### 2.3. Measures

#### 2.3.1. Commuting Patterns

Participants were asked to reflect on the previous month and report the average number of times per week in the last month that they walked, biked, drove, and took public transportation (where available) to and from work. For each mode of travel, a dichotomized variable was created to indicate no travel by the mode of travel or travel via the mode one or more times per week. Public transportation ridership was only considered among those who had public transit available to them as determined by self-report of public transit availability in their community (*n* = 748). Respondents also indicated the perceived number of minutes it would take them to walk and bike to work using one item for each mode.

#### 2.3.2. Demographics and Health Outcomes

Participants reported their age, sex, race/ethnicity (collapsed into non-Hispanic White, non-Hispanic Black, and other racial/ethnic groups), and income level. Participants responded (yes/no) if they had any cardiovascular/pulmonary disease (heart disease, high blood pressure, elevated cholesterol, and chronic obstructive pulmonary disease), metabolic disease (diabetes, liver, or thyroid disease), musculoskeletal disease (arthritis, osteoporosis), or depression, and a total number of chronic diseases was calculated. Diseases were then collapsed into the four categories and dichotomized (e.g., yes/no for reporting a metabolic disease). Individuals were also asked to report their height and weight for body mass index (BMI) calculations (weight in kg/(height in meters)^2^). Respondents also rated their current health status from 1 (poor) to 5 (excellent). To determine if individuals were meeting current physical activity recommendations (at least 150 minutes/week of moderate intensity physical activity) [46] from their AC participation, the number of trips per week walking and biking were multiplied by the amount of time they reported for a walk or bike trip to work. The total number of minutes of AC time was calculated and was then dichotomized as meeting recommendations via active commuting (+150 minutes/week of active travel to work) or not meeting recommendations.

#### 2.3.3. Perceived Health Benefits of AC

Respondents indicated their agreement with eight statements related to physical or mental health benefits of AC (e.g., AC helps me control my weight; AC can help me to relieve stress) using a 7-point Likert scale (1 = completely disagree to 7 = completely agree). A summed score was computed for all 8 items. This scale was based on a previously-tested measure [47] and showed excellent reliability in the present sample (*α* = 0.89).

### 2.4. Statistical Analyses

Basic descriptives and frequencies were used to describe the sample. To examine the primary aim, for each mode of travel, separate univariate logistic regression models were used to predict the likelihood of walking, biking, driving, and public transit use at least once per week according to demographics and health-related factors (age, sex, income, race/ethnicity, chronic disease presence, perceived health status, and perceived health benefits of AC). Factors significantly associated with walking, biking, driving, and use of public transit were examined simultaneously in four multivariate logistic regression models and the Nagelkerke *R* ^2^ was calculated for each of the full models to examine the factors associated with each mode of travel. To address the secondary aim, the likelihood of meeting physical activity recommendations via AC was examined via univariate logistic regression with the same demographics and health-related factors and then a full model with significant factors was performed. All analyses were performed using SPSS 20.0 (Armonk, NY) and significance levels were set at *P* < 0.05. 

## 3. Results

The demographics of the sample are shown in Table 1. Participants were primarily non-Hispanic White (92.1%), female (68.3%), and had a high (over $60,000) income level (63.2%). The mean age of respondents was 43.8 years (s.d. = 11.4) and slightly more than half of respondents were overweight (31.5%) or obese (19.5%). Most individuals (78.3%) reported driving to work one or more times/week and 20.3% reported using public transit, though relatively few reported walking (7.3%) or biking (14.4%) one or more times/week. Among those traveling using active methods, 9.6% met physical activity recommendations via AC.

### 3.1. Walking to Work One or More Times/Week

Univariate influences on walking to work at least once per week are found in the first columns of Table 2. Age was negatively related to being a walker (OR = 0.97, 95% CI = 0.95–0.99). Those from “other” racial/ethnic groups were more likely to walk (OR = 2.99, 95% CI = 1.44–6.25) compared to non-Hispanic Whites. Better perceived health status was associated with being a walker (OR = 1.64, 95% CI = 1.24–2.17) and being in the obese weight category was associated with being a non-walker (OR = 0.46, 95% CI = 0.23–0.93). The full model of significant correlates resulted in a Nagelkerke *R* ^2^ of 0.07, with race (“other” racial/ethnic group OR = 2.91, 95% CI = 1.34–6.31), age (OR = 0.97, 95% CI = 0.95–0.99), and perceived health status (OR = 1.60, 95% CI = 1.17–2.20) as significant predictors of walking to work. 

### 3.2. Biking to Work One or More Times/Week

The univariate influences for biking to work are found in Table 2. Similar to those walking to work, age was negatively associated with being a biker (OR = 0.93, 95% CI = 0.92–0.95) and females were less likely to bike to work than males (OR = 0.31, 95% CI  = 0.22–0.43). Higher income status was associated with being a non-biker, with both the $30,000–$60,000 groups (OR = 0.38, 95% CI = 0.20–0.71) and the $60,000 and up group (OR  = 0.31, 95% CI = 0.18–0.60) less likely to bike to work than the lowest income group (<$30,000/year). Those from “other” racial/ethnic groups were more likely to bike (OR = 3.04, 95% CI = 1.65–5.59) compared to non-Hispanic Whites. A greater number of chronic diseases was associated with being a non-biker (OR = 0.78, 95% CI = 0.65–0.94) while better perceived health status was associated with biking (OR = 1.98, 95% CI = 1.59–2.45). Those reporting metabolic disease (OR  = 0.26, 95% CI = 0.11–0.60), overweight (OR = 0.58, 95% CI = 0.39–0.85), and obese status (OR = 0.26, 95% CI = 0.14–0.47) were less likely to report biking. Those with greater perceived health benefits of AC were more likely to be bikers (OR = 1.05, 95% CI = 1.03–1.08). A full multivariate model revealed a Nagelkerke *R* ^2^ of 0.27, with race (“other” racial/ethnic group OR = 2.40, 95% CI = 1.09–5.30), age (OR  = 0.93, 95% CI  = 0.92–0.95), income ($30,000–60,000/year OR = 0.31, 95% CI = 0.14–0.69; <$60,000/year OR = 0.36, 95% CI = 0.16–0.78), perceived health status (OR = 1.95, 95% CI = 1.47–2.61), and AC health beliefs (OR = 1.05, 95% CI = 1.02–1.08) as significant predictors.

### 3.3. Driving to Work One or More Times/Week

Univariate analyses for driving to work are displayed in Table 2. Older age was associated with driving one or more times per week (OR = 1.04, 95% CI = 1.03–1.06), and those from “other” racial/ethnic groups were less likely to be drivers compared with non-Hispanic Whites (OR = 0.21, 95% CI = 0.11–0.39). Females were more likely to report driving compared to males (OR = 2.88, 95% CI = 1.98–4.19). A greater number of chronic diseases (OR = 2.13, 95% CI = 1.72–2.64) and poorer perceived health status (OR = 0.63, 95% CI = 0.49–0.80) were also associated with being a driver. Reporting cardiopulmonary disease (OR = 3.37, 95% CI = 2.20–5.16), metabolic disease (OR = 4.20, 95% CI = 2.11–8.37), musculoskeletal disease (OR  = 3.29, 95% CI = 1.70–6.37), depression (OR = 2.75, 95% CI = 1.64–4.63), or being overweight (OR = 2.59, 95% CI = 1.40–4.79) was associated with being a driver. The full model resulted in a Nagelkerke *R* ^2^ value of 0.12, with race (“other” racial/ethnic group OR = 0.26, 95% CI = 0.13–0.55), age (OR  = 1.03, 95% CI = 1.01–1.05), and perceived health status (OR = 0.62, 95% CI = 0.46–0.84) as significant predictors.

### 3.4. Public Transportation One or More Times/Week

Table 2 outlines the univariate influences on public transportation use. Similar to walking and biking, younger age was associated with being a public transit rider to work (OR = 0.97, 95% CI = 0.95–0.99). Those reporting a higher income were less likely to report public transit ridership compared with those at the lowest income level (OR = 0.46, 95% CI  = 0.23–0.93). Females were less likely to be transit riders compared to males (OR = 0.60, 95% CI = 0.41–0.87), and those from “other” racial/ethnic groups were more likely to use transit compared with non-Hispanic Whites (OR = 2.32, 95% CI = 1.17–4.60). There were no health-related variables significantly associated with public transit use. The full model had a Nagelkerke *R* ^2^ of 0.06, with age (OR = 0.97, 95% CI = 0.95–0.99) as a significant predictor.

### 3.5. Meeting Physical Activity Recommendations via Active Commuting

The univariate analyses examining influences on meeting physical activity recommendations via AC are found in Table 2. Younger age was associated with meeting recommendations from AC (OR = 0.93, 95% CI = 0.91–0.95). Those earning $30,000–60,000/year (OR = 0.32, 95% CI = 0.17–0.63) and greater than $60,000/year (OR = 0.24, 95% CI  = 0.13–0.45) were less likely to meet recommendations via AC compared with those in lower income groups. Females were less likely to meet recommendations relative to males (OR = 0.28, 95% CI = 0.19–0.41) and those in the “other” racial/ethnic group were more likely to meet recommendations compared with non-Hispanic Whites (OR = 3.41, 95% CI = 1.77–6.57). Participants reporting more chronic diseases (OR = 0.76, 95% CI = 0.61–0.96) and specifically cardiopulmonary (OR = 0.58, 95% CI = 0.34–0.97), metabolic (OR = 0.58, 95% CI = 0.35–0.97), or musculoskeletal disease (OR = 0.34, 95% CI = 0.14–0.85) were less likely to meet recommendations. Overweight (OR = 0.62, 95% CI = 0.39–0.97) and obese (OR = 0.35, 95% CI = 0.18–0.67) individuals were also less likely to meet recommendations from AC. Finally, those who perceived greater health benefits of AC (OR = 1.04, 95% CI = 1.01–1.07) and more positive personal health status (OR = 1.64, 95% CI = 1.28–2.11) were more likely to meet recommendations. The full model resulted in a Nagelkerke *R* ^2^ of 0.26, with perceived health status (OR  = 1.53, 95% CI = 1.11–2.12), age (OR = 0.94, 95% CI = 0.92–0.96), higher income ($30,000–60,000/year OR = 0.29, 95% CI = 0.13–0.68 and <$60,000/year OR = 0.26, 95% CI = 0.11–0.59), and female gender (OR = 0.24, 95% CI = 0.15–0.39) as significant predictors.

## 4. Discussion

This study revealed a number of relationships between health-related outcomes and mode of travel to work. For the active transportation modes, there were a number of significant health-related influences while poorer health outcomes were associated with the more passive forms of travel. Understanding this relationship between choice of travel mode to work and health outcomes allows for the development of interventions to promote AC, with considerations for some of the health-related concerns addressed with this study. Among those who were active enough using active transportation modes, the noted health-related influences have considerable implications for public health. 

There is clear evidence that physical activity participation [3], and specifically AC, can result in positive health outcomes [20, 21, 23, 25]. Other studies have also highlighted how continued physical activity participation and lifestyle choices can help to manage diabetes [48, 49] or cardiovascular disease [50–52] and help with cancer survivorship [53, 54]. In the current study, those with cardiopulmonary, metabolic and musculoskeletal disease, and depression were more likely to choose the more passive mode of travel, driving. Although these individuals are already impacted with these chronic conditions, the evidence suggests that increased physical activity can help with management of these diseases, and active travel could possibly contribute to achieving current public health recommendations for physical activity [46]. 

A small portion of participants in the current study reported meeting physical activity recommendations based on active transportation alone. Recent models attempting to understand the influences on physical activity participation have indicated that active transportation is an often overlooked method of accumulating recommended amounts of physical activity [55, 56]. Data from Kaczynski and colleagues indicated that adults reporting walking or biking for transportation at least once per week was associated with meeting physical activity recommendations [45], similar to a study by Berrigan and colleagues [57]. Other studies by Yang et al. [42] and Sahlqvist et al. [44] found a positive relationship between AC and daily physical activity participation and other studies have confirmed that more time spent in cars is associated with less time for physical activity participation [58]. Using public transportation can often serve as a catalyst for encouraging physical activity; for example, studies have shown that using public transportation is associated with significant walking to and from transit [43, 59], and many individuals meet current physical activity recommendations through active transport to and from transit locations [43, 60]. Therefore, where feasible, public health campaigns may wish to encourage transit use over vehicular commuting, and this strategy and behavior may be more palatable to a large segment of the population who eschew the idea of biking and walking to work.

The majority of literature has focused on travel to work as a collapsed variable (e.g., walking and biking combined) and limited research as examined how specific modes of travel are related to health outcomes. A study examining the relationship between commuting and health outcomes in Sweden also found poorer health outcomes (sleep quality, everyday stress, and frequent illness) and perceived health associated with commuting via car [61]. Hemmingsson and colleagues [62] found that commuting via bicycle (but not walking) was associated with improved diabetes biomarkers among obese women. Frank and colleagues [63] also noted an elevated risk of obesity as time spent in cars increased, and time walking was associated with less obesity. Another study noted that switching to commuting by public transportation instead of a car increased energy expenditure and decreased body fat [64]. Zheng [65] also noted that those commuting by public transportation were 44.6% less likely to be overweight due to an increase in walking or biking associated with transit use. The present study adds to our understanding of how a variety of health-related factors and other demographic indicators are associated specifically with each of walking, biking, driving, and use of public transportation to work.

In the current study, race/ethnicity was a significant influence in several analyses. Limited research has addressed racial/ethnic differences in AC among adults, though a number of studies have noted different trends among youth traveling to school [66–68]. There is some evidence to suggest that there are differences in active transportation rates among adults [42, 57, 69] though there is little mode-specific information available. Other studies have indicated that rates of leisure time physical activity are lower in ethnic minority groups, and household or occupational activity is higher [70–74]. This would suggest that some social or cultural differences associated with AC may exist that have not been well-explored. The results of this study add to the limited research in this area regarding mode choice to work and race/ethnicity.

Employee health is often a significant concern for employers, with absenteeism, productivity, and health insurance benefits representing substantial costs. Interventions and strategies targeting physical activity participation in worksite settings have noted positive cost effectiveness outcomes associated with behavior change among employees [75–79]. Therein, there is notable interest in understanding all types of physical activity participation and influences among employed adults. As noted above, transportation related activity has many documented benefits and may be a time-effective approach to including physical activity into one's day for busy, working adults. Lachapelle and Frank [60] noted that employer-sponsored transit passes were associated with increased physical activity participation and other research has shown that workplace supports for AC can be a significant influence on participation [37, 80]. Employers may benefit from developing supporting policies or programs to encourage active forms of travel with the long term goal of reduced chronic disease morbidity and mortality among employees, for example, enacting policies regarding a flexible dress code to allow for active travel or develop incentive programs to reward employees who walk or bike to work.

This study also revealed that older adults and those in poorer health are less likely to actively travel to work. These findings present some challenges for practitioners looking to target this behavior within this population. Additional research may be needed to determine what the specific barriers to AC are for these populations in order to effectively improve behavior. For example, some research has shown that sidewalks, a key piece of the active transportation infrastructure in many communities, are more lacking in quality in lower income areas [81]. This may be especially problematic for older adults and persons with mobility impairments. Additionally, intervention strategies targeting AC could draw on the abundance of evidence found in physical activity interventions tailored for older adults or clinical populations. Some of the strategies that could be translated into an intervention targeting older employees or employees with chronic conditions could include use of social support from coworkers or family, improving self-efficacy for AC, providing education on the benefits of AC, enlisting healthcare providers' advice for increasing physical activity, use of self-monitoring and self-regulatory skills, and creating activity-friendly environments and policies [82–86]. Behavior-change theory should also be applied to target known mediators of AC (e.g., self-efficacy, outcome expectations) and improve the effectiveness of interventions [87]. 

Although this study yielded a number of important insights into the relationship between mode of transportation and health outcomes, there are some noteworthy limitations. The convenience sampling strategy used in this study may not have recruited respondents who are representative of the larger population, though rates of AC were similar to many of the other studies cited. Though the response rate was also low, it was calculated conservatively, as we were unable to determine how many of our email invitations were channeled into “junk/spam” mailboxes, thereby remaining unread by potential respondents. The cross-sectional study also limits our ability to draw causal inferences between AC and health outcomes. Although significant evidence has indicated the importance of environmental-level variables, these were not examined in the current study; however additional analyses with these variables are found elsewhere [80]. It should also be noted that dichotomizing the mode of travel as none versus one or more trips per week may have resulted in frequent walkers and bikers being categorized with those who walk or bike infrequently. Although those who actively commute even a limited amount of time are likely to be more similar to employees who walk or bike to work a lot than those who do not actively commute at all, these categorization decisions could present some challenges with interpreting our findings. Finally, we used self-reported, unvalidated measures of AC behavior and health outcomes, which have limited objectivity. Future studies should use multiple measures of behavior and health outcomes to enhance the validity and reliability of the data.

Despite these limitations, this study contributes to the literature on how mode of travel to work is associated with health outcomes. Given the documented benefits associated with participation in physical activity and specifically AC, there is merit in examining the possible role these behaviors can contribute to reducing morbidity and mortality from the leading chronic diseases and associated healthcare expenditures. Community design and environmental supports, along with worksite programs and policies, can influence travel choices and should be considered as targets for interventions for improving population-level health.

## Figures and Tables

**Table 1:** Characteristics of the sample (*n* = 1234).

Variable	*n* (%)	Mean (SD)
Demographic		
Age		43.76 (11.44)
Sex		
Male	327 (31.7)	
Female	706 (68.3)	
Income level		
<$30 K/year	55 (5.5)	
$30–60 K/year	309 (31.2)	
>$60 K/year	626 (63.2)	
Race/ethnicity		
Non-Hispanic White	941 (92.1)	
Non-Hispanic Black	33 (3.2)	
All other racial/ethnic groups	48 (4.8)	
Health related		
Number of chronic disease		0.64 (1.01)
Reporting chronic disease		
CV pulmonary disease	286 (21.8)	
Metabolic disease	133 (10.2)	
Musculoskeletal disease	120 (9.2)	
Depression	170 (13.0)	
Body mass index		
Normal weight	460 (49.0)	
Overweight	296 (31.5)	
Obese	183 (19.5)	
Psychological		
Perceived health status (range 1–5)		3.68 (0.81)
Perceived health benefits (range 8–56)		44.04 (7.91)
Mode of travel to work		
Walking one or more time/week	95 (7.3)	
Biking one or more time/week	188 (14.4)	
Driving one or more time/week	1026 (78.3)	
Public transit use one or more time/week	152 (20.3)	

AC: active commuting.

**Table 2:** Univariate influences on walking, biking, driving, and taking public transit to work, meeting physical activity recommendations via active transport modes.

Variable	Walking to work at least 1 time/week		Biking to work at least 1 time/week		Driving to work at least 1 time/week		Public transit to work at least 1 time/week		Meeting physical activity recommendations via active transport modes	
	OR	95% CI	OR	95% CI	OR	95% CI	OR	95% CI	OR	95% CI
Demographic variables										
Age	0.97**	0.95–0.99	0.93***	0.92–0.95	1.04***	1.03–1.06	0.97**	0.95–0.99	0.93***	0.91–0.95
Income										
<$30,000/year (referent)	1		1		1		1		1	
$30,000–60,000/year	0.71	0.29–1.71	0.38**	0.20–0.71	1.1	0.52–2.32	0.67	0.32–1.41	0.32***	0.17–0.63
>$60,000/year	0.64	0.26–1.41	0.33***	0.18–0.60	1.94	0.94–4.05	0.46*	0.23–0.93	0.24***	0.13–0.45
Sex: female (male referent)	0.85	0.54–1.35	0.31***	0.22–0.43	2.88***	1.98–4.19	0.60**	0.41–0.87	0.28***	0.19–0.41
Race										
Non-Hispanic White (referent)	1		1		1		1		1	
Non-Hispanic Black	0.73	0.17–3.13	0.33	0.08–1.38	0.91	0.31–2.64	1.65	0.63–4.36	0.19	0.04–1.92
All other racial/ethnic groups	2.99**	1.44–6.25	3.04***	1.65–5.59	0.21***	0.11–0.39	2.32*	1.17–4.60	3.41***	1.77–6.57
Health-related variables										
Number of chronic disease	0.96	0.77–1.18	0.78*	0.65–0.94	2.13**	1.72–2.64	1.03	0.88–1.22	0.76*	0.61–0.96
Reporting chronic disease (no disease as referent)										
CV pulmonary disease	0.98	0.59–1.63	1.37	0.92–2.04	3.37***	2.20–5.16	0.88	0.58–1.33	0.58*	0.34–0.97
Metabolic disease	0.58	0.25–1.35	0.26***	0.11–0.60	4.20***	2.11–8.37	1.23	0.67–2.25	0.58*	0.35–0.97
Musculoskeletal disease	1.1	0.52-2.33	1.73	0.91–3.28	3.29***	1.70–6.37	1.21	0.66–2.21	0.34*	0.14–0.85
Depression	1.28	0.72–2.28	1.21	0.78–1.87	2.75***	1.64–4.63	0.68	0.44–1.09	1.14	0.67–1.93
Body mass index										
Normal weight	1		1		1		1		1	
Overweight	0.77	0.47–1.27	0.58**	0.39–0.85	1.34	0.88–2.04	0.98	0.64–1.51	0.62*	0.39–0.97
Obese	0.46*	0.23–0.93	0.26***	0.14–0.47	2.59**	1.40–4.79	0.77	0.44–1.34	0.35**	0.18–0.67
Psychological variables										
Perceived health status^a^	1.64**	1.24–2.17	1.98***	1.59–2.45	0.63***	0.49–0.80	0.87	0.69–1.11	1.64***	1.28–2.11
Perceived health benefits of AC	1.03	0.99–1.06	1.05***	1.03–1.08	0.98	0.95–1.01	0.99	0.97–1.02	1.04**	1.01–1.07

Note: *P < 0.05, **P < 0.01, ***P < 0.001, ^a^scale ranges 1 (poor) to 5 (excellent), and AC: active commuting.
